# Heavy Metal in Paddy Soil and its Bioavailability in Rice Using In Vitro Digestion Model for Health Risk Assessment

**DOI:** 10.3390/ijerph16234769

**Published:** 2019-11-28

**Authors:** Nur Syahirah Zulkafflee, Nurul Adillah Mohd Redzuan, Zanjabila Hanafi, Jinap Selamat, Mohd Razi Ismail, Sarva Mangala Praveena, Ahmad Faizal Abdull Razis

**Affiliations:** 1 Department of Food Science, Faculty of Food Science and Technology, Universiti Putra Malaysia, Serdang 43400 UPM, Selangor, Malaysia; nursyahirahzulkafflee@gmail.com (N.S.Z.); dillaredzuan94@gmail.com (N.A.M.R.); sjinap@gmail.com (J.S.); 2 Department of Environmental and Occupational Health, Faculty of Medicine and Health Sciences, Universiti Putra Malaysia, Serdang 43400 UPM, Selangor, Malaysia; zanjabila@gmail.com (Z.H.); smpraveena@upm.edu.my (S.M.P.); 3 Laboratory of Food Safety and Food Integrity, Institute of Tropical Agriculture and Food Security, Universiti Putra Malaysia, Serdang 43400 UPM, Selangor, Malaysia; 4 Laboratory of Climate-Smart Food Crop Production, Institute of Tropical Agriculture and Food Security, Universiti Putra Malaysia, Serdang 43400 UPM, Selangor, Malaysia; razi@upm.edu.my; 5 Laboratory of Molecular Biomedicine, Institute of Bioscience, Universiti Putra Malaysia, Serdang 43400 UPM, Selangor, Malaysia

**Keywords:** bioaccumulation, heavy metal, hazard quotient, lifetime cancer risk

## Abstract

Rice ingestion is one of the major pathways for heavy metal bioaccumulation in human. This study aimed to measure the heavy metal content of paddy soils and its bioavailability in paddy grain in order to assess the health risk. In total, 10 rice samples (50 g each) of paddy plants were harvested from the Selangor and Terengganu areas of Malaysia to assess the bioavailability of heavy metal (As, Cd, Cu, Cr, and Pb) using the in vitro digestion model of Rijksinstituut voor Volksgezondheid en Milieu. The bioavailability of heavy metal concentrations in rice samples were analyzed using Inductively Coupled Plasma Mass Spectrometry (ICP-MS). The findings showed the bioavailability of heavy metal concentrations was decreased in the order Cr > Cu > Pb > As > Cd. Chromium was found to be the most abundant bioavailable heavy metal in cooked rice, which was the result of its high content in paddy soil. Hazard Quotient values for the bioavailability of the heavy metal studied were less than one indicating no non-carcinogenic health risks for adults and children. Meanwhile, the total Lifetime Cancer Risk exceeded the acceptable value showing a potential of carcinogenic health risk for both adults and children. The application of in vitro digestion model in assessing bioavailability of heavy metal produces a more realistic estimation of human health risks exposure. However, a regular monitoring of pollution in Selangor and Terengganu areas is crucial since the exposure of heavy metals through rice consumption poses the potential non-carcinogenic and carcinogenic health risk to the local residents.

## 1. Introduction

Human activities in meeting the needs of life have an impact on the environment. This impact can either be positive or negative. One negative impact can be a decrease in environmental quality, including quality of the land, such as a decline in soil quality due to pollution by waste produced by humans. The accumulation of heavy metals in agricultural land is a growing concern for the general public as it has detrimental effects on soil ecosystems and potential health risks [1]. International agencies such as the Food and Agriculture Organization (FAO) and the World Health Organization (WHO) now adhere to the criteria of pollutant truth in agricultural products [2]. Heavy metals are found in all areas of major pollution in food [3]. Heavy metals such as arsenic, cadmium, and mercury are major concerns in soil and food pollution, significantly in the rice cultivation system, due to their toxicity. Mercury, copper, lead, cadmium and arsenic are types of dangerous heavy metals that often contaminate the soil and make them dangerous substances for plants [4].

Food consumed by humans is mostly a product of agriculture, and agricultural activities may be the pollutant source for soil contamination, while rice consumption is a staple food source for human consumption. If the soil is contaminated with heavy metals, then the quality of food can be bad and even harmful to humans. Arsenic is an important component for some animals and plants, but arsenic is better known as a type of poison rather than its capacity as an essential component. This compound is found in the form of oxide and its toxicity depends on the shape of its chemical structure [5]. Meanwhile, cadmium is a metal that is toxic to the human body. The color is bluish and if it enters the kidney it can accumulate and cause the kidneys to become dysfunctional [6]. The structure of the edible part of the plant such as the grains can cause the direct transfer of metal pollutants to humans [4]. Harvested rice is easily exposed to heavy metal pollutants from agricultural crops planted with contaminated water, metal-based fertilizers and pesticides, completely at different process levels (puncture, polishing, and bleaching), transport, collection methods and storage of the plants [7].

Bio-accessibility works as a term that describes the extent of the absorption of the contents in the food into the body [8]. The bio-accessibility of heavy metals is a major method in assessing their danger to human well-being. Digestion can be carried out in vitro using enzymatic processes [9]. The enzymatic process occurs in the reshuffle of protein and fat [10]. An in vitro digestion is a digestion that occurs outside the digestive organ or is performed in a laboratory. This model is widely used to study the structural changes, digestibility, and release of food components under simulated gastrointestinal conditions [11]. The Rijksinstituut voor Volksgezondheid en Milieu (RIVM) in vitro digestion model is an example of a bioavailability method used in rice studies by rice ingestion where the model is quite similar to the physiological condition of the human body. However, its results are often different to those found using in vivo models because of the difficulties in accurately simulating the highly complex physicochemical and physiological events occurring in animal and human digestive tracts [11]. The most frequently used biological molecules included in the digestion model are digestive enzymes (pancreatin, pepsin, trypsin, chymotrypsin, peptidase, α-amylase, and lipase), bile salts, and mucin [7,8]. In vitro digestion according to Omar et al. [7] is the most effective way for chemical activity determination and is a cost saving tool.

There are three methods of determining heavy metals such as acid digestion methods, metal fractionation and an in vitro digestive model [8]. There is a limit to the determination of heavy metals using the acid digestion and fractionation methods due to the amount of heavy metal concentrations that do not represent the properties of the heavy metals and their toxicity [8]. However, the in vitro digestive model is preferred over acid digestion and the fractionation method in the determination of heavy metals [8]. This is due to the right yield in a short, easy and inexpensive manner when using the in vitro digestion model as well as a reduction in the use of experimental animals and allowing replications of samples.

According to the United States Environmental Protection Agency (USEPA) 2012 [12], human health risk assessments (HRA) due to environmental hazard exposures are considered as characterization of the adverse health effects of humans. Scientific, engineering and statistical tools are required in identifying and measuring hazards, determining the potential for subsequent exposure paths using existing information to calculate numerical values that represent potential risks. Human health risk assessments involve four steps: hazard identification, dose–response assessment, exposure assessment, and risk characterization [12]. Carcinogenic and non-carcinogenic elements are classified under health risk assessments [13]. There are previous studies involving the determination of heavy metal contamination, but the studies have been focused on the total heavy metal concentrations, in which the overall hazard risk to human health is not represented [7]. The USEPA’s public health risk assessments is aimed at protecting all potentially affected populations, including subpopulations on the basis of gender, nutritional status, genetic predisposition, and life stages (e.g., childhood, pregnancy, old age) that might be more susceptible to toxic effects or that are highly or disproportionately exposed (e.g., children, ethnic groups) [12]. Children have been identified as a special population to consider in risk assessment because their health risks can differ from those of adults as a result of their immature physiology, metabolism, and different levels of exposure [12].

Based on a previous study, the heavy metal content in paddy soils and rice grains was not well documented and there was limited information regarding the health risk assessment for rice consumption among the local population. Therefore, the current study, which is related to the bio-accessibility of heavy metal in rice grain can provide an understanding of hazard-assessments for humans caused by heavy metals. Referring to the Fourteenth Schedule (Regulation 38), which is supported by Malaysian Food Regulations (1985), consumer rights are maintained for the permissible level of heavy metals. Food standards such as FAO/United Nations Commission on Codex Alimentarius (CAC) 1984 is to ensure honest practice in consumer trade and safety [14,15]. Therefore, the bio-accessibility of heavy metal concentrations was compared with the permissible levels of heavy metal expressed within the Malaysian Food Regulations (1985) and the Food and Agriculture Organization/United Nations agency CAC 1984.

This study was designed to measure the content of heavy metals (Pb, Cd, Cu, Cr and As) in soils and to assess the health risk towards the bio-accessibility of heavy metals in rice grains for local residents in Selangor and Terengganu since rice is a staple food in Malaysia.

## 2. Materials and Methods

### 2.1. Sampling Area

The surface soil and paddy plant samples were collected from paddy planting areas in Selangor and Terengganu, Malaysia. Selangor is one of the major rice producing areas of Malaysia located on the west of Peninsular Malaysia, overlooking the Straits of Malacca while Terengganu is situated in the eastern Peninsular of Malaysia, and is bordered in the Northwest by Kelantan, the Southwest by Pahang, and the east by the South China Sea. Sampling of paddy plants were conducted in May till August 2018 simply before harvest time. Three paddy planting areas in Selangor (Sabak Bernam, Sekinchan and Tanjung Karang) within the area from latitude 3°24′59.99″ N to 3°46′11.28″ N and from longitude 100°59′16.44″ E to 101°10′60.00″ E and one area in Terengganu (Besut) from latitude 5°49′44.44″ N and longitude 102°33′8.57″ E were chosen indiscriminately. Three plots, which were 1000 m^2^ each were randomly selected for sampling activities at each sampling area. The paddy plant was uprooted with the soil from every plot.

Surface soil samples were taken from 0 to 30 cm depth with a soil hand auger, stored in clean bags and labelled properly. The samples were labelled representing the areas studied, SBP1, SBP2, and SBP3 for Sabak Bernam plots 1, 2 and 3; SKP1, SKP2, and SKP3 for Sekinchan plots 1, 2 and 3; TKP1, TKP2 and TKP3 for Tanjung Karang plots 1, 2 and 3 and BP1 for Besut plot 1, respectively. In the laboratory, the soil samples were dried at room temperature before being ground and sieved with a 250 μm sieve, prior to analysis. The heavy metals within the soil were extracted using ammonium acetate (pH 7) [16,17]. Ten grams of dried soil was weighed into a Kartell bottle, then 50 mL of 1.0 M NH_4_CH_3_OO (pH 7) was added and the mixture were shaken for 1.5 h, followed by centrifugation at 3000 rpm for half an hour. The samples were then filtered using a 0.45 μm syringe filter (Millipore, Merck, Kenilworth, New Jersey, USA). Heavy metal components of As, Cd, Cu, Cr and Pb were analysed in the samples by using Inductively Coupled Plasma-Mass Spectrometry (ICP-MS) (Perkin Elmer Elan DRC-e, Waltham, Massachusetts, USA). Nevertheless, the soil samples did not undergo the in vitro digestion step because soil is not a food component but it acts as a medium for plant growth.

### 2.2. Sample Preparation of Paddy Grains Before Undergoing In Vitro Digestion

In total, 10 samples of grain were prepared, 50 g from each sampling plot. The samples were washed rapidly three times with deionised water. The samples were then cooked with 100 mL deionised water in a ratio of 1:2 according to Omar et al. [7]. A drying oven (Memmert Oven, Schwabach, Germany) was used to dry the cooked rice samples at 65 °C for 48 h before being ground with a pestle and mortar. The grinded samples were sieved using a 0.25 mm mesh sieve (No. 60 mesh sieve) and kept in sealed plastic bags at 4 °C before undergoing digestion steps using the Rijksinstituut voor Volksgezondheid en Milieu (RIVM) in vitro digestion model [7]. In vitro digestion models are widely used to study the structural changes, digestibility, and release of food components under simulated gastrointestinal conditions [8].

The mouth, stomach and small intestine models play a main role in the RIVM in vitro digestion models. Potassium chloride (KCl), potassium thiocyanate (KSCN), sodium dihydrogen phosphate (NaH_2_PO_4_), disodium sulfate (Na_2_SO_4_), sodium chloride (NaCl), sodium bicarbonate (NaHCO_3_), urea, a-amylase, uric acid and mucin were used to simulate artificial saliva in the mouth. Additionally, sodium chloride (NaCl), sodium dihydrogen phosphate (NaH_2_PO_4_), calcium chloride dehydrate (CaCl_2_∙2H_2_O), ammonium chloride (NH_4_Cl), hydrogen chloride (HCl), glucose, glucuronic acid, urea, glucosamine hydrochloride, Bovine Serum Albumin (BSA), pepsin and mucin were used to represent gastric juice inside the stomach. Meanwhile, NaCl, NaHCO_3_, potassium dihydrogen phosphate (KH_2_PO_4_), KCl, magnesium chloride (MgCl_2_), HCl, CaCl_2_∙2H_2_O, BSA, pancreatin and lipase were used as duodenal juice. Lastly, NaCl, NaHCO_3_, KCl, HCl, urea, CaCl_2_∙2H_2_O, BSA and bile were used for bile juice [8]. All the chemicals used were prepared on the day of the analysis.

### 2.3. Heavy Metal Analysis

Inductively Coupled Plasma-Mass Spectrometry (ICP-MS) (Perkin Elmer Elan DRC-e, Waltham, Massachusetts, USA) was used to analyse the heavy metal content in the samples. In order to maintain the quality, 10% nitric acid (HNO_3_) was used to soak all the lab equipment. A standard solution was run together with the blank sample in every 10th sample and rice samples were run in triplicate together with Certified Reference Material (CRM) IRMM 804 in the range of 90.5% to 102.4% as reported by Omar et al. [7].

### 2.4. Statistical Analysis

Statistical analysis of the data was performed using Minitab (Version 17.0, Minitab Pennsylvania, State College, Pennsylvania, USA) by one-way analysis of variance (ANOVA) to determine the difference between the concentration of heavy metals in the cooked rice samples and Hazard quotient (HQ) and lifetime cancer risk (LCR) values (representing HRA). The HRA values were calculated based on the equation provided by USEPA 2012 [12]. The typical rice Ingestion Rates (IR) used for adults and children were 600 g/day and 198.4 g/day, respectively [7].

### 2.5. Hazard Quotient (HQ) and Lifetime Cancer Risk (LCR)

Hazard Quotient (HQ) is an example model used to analyze human non-carcinogenic risk for local inhabitants. HQ is able to characterize the health risk of non-carcinogenic adverse effects due to exposure to toxicants by which the reference dose (RfD) is the estimated allowable dose for humans via daily exposure [12,14]. If HQ < 1, adverse health effects would be unlikely to be experienced whereas if HQ ≥ 1, potential non-carcinogenic effects would occur [12]. Lifetime cancer risk (LCR) has been determined to estimate the incremental probability of an individual developing cancer over a lifetime. For example, a LCR of 10^−4^ indicates a probability of 1 in 10,000 individuals developing cancer. The total CR (CRt) from all carcinogens is summed by assuming additive effects, if the multiple carcinogenic elements are present. The method of estimating LCR was provided in the USEPA Region III Risk-Based Concentration [12]. The equations used for the calculation of the hazard quotient (HQ) and lifetime cancer risk (LCR) are shown below. HQ (Equation (1)) denotes a ratio of average daily dose (ADD) to the reference dose (RfD), whereas, LCR was calculated for carcinogenic health risk by multiplying the average daily dose (ADD) in mg/kg·day over a lifetime with a cancer slope factor (CSF) according to Equation (2) [12].

HQ = ADD (mg/kg·day)/RfD (mg/kg·day)(1)

where ADD (mg/kg·day) is a parameter used to calculate the oral exposure dosage during a specific period and expressed into a daily dose per unit body weight and RfD (mg/kg·day) is the estimated maximum permitted dose for humans through daily exposure.

LCR = ADD (mg/kg·day) × CSF (mg/kg·day)^−1^ (2)

where ADD (mg/kg·day) is a parameter used to calculate the oral exposure dosage during a specific period and expressed as a daily dose per unit body weight and CSF (mg/kg·day)^−1^ is a cancer slope factor.

## 3. Results and Discussion

### 3.1. Concentrations of Heavy Metal in Paddy Soil

The concentrations of heavy metal in paddy soils for Sabak Bernam, Sekinchan, Tanjung Karang and Besut are shown in Table 1. In Sabak Bernam area, plot 1 showed the highest concentration of chromium (Cr) while plot 2 and plot 3 showed the highest concentration of lead (Pb). Meanwhile, all paddy soil samples in Tanjung Karang (TKP1, TKP2 and TKP3), Sekinchan (SKP1, SKP2 and SKP3) and Besut (BP1) showed high concentrations of the heavy metal element Pb, respectively. Hence, Besut plot 1 (BP1) gave the highest readings for heavy metal concentrations of Pb among all samples with a range from 2.3799 mg/kg to 2.6399 mg/kg. However, all elements (Cu, Cd, Cr, Pb and As) in paddy soils for all studied areas fell below the maximum allowable concentration based on standards recommended by the Dutch Soil Remediation Circular 2009 [17] and European Standards Agriculture Soils [18]. The ranking order of heavy metal occurrence in paddy soils of Sabak Bernam, Sekinchan, Tanjung Karang and Besut was Pb > Cr > Cu > As > Cd, which stipulated that lead (Pb) and chromium (Cr) were the most abundant heavy metals of those studied in all paddy soil samples while cadmium was the lowest. The lowest concentration of Cd in paddy soils was consistent with previous studies conducted in Malaysia [1,16,19,20,21,22]. The Cd content decreased while Pb increased indicating that Pb had a higher affinity and stability to the organic matter of soil compared to the Cd element [10]. However, different findings were found by Habibah et al. [23] in paddy soils from Penang and Kedah in Malaysia where Cd concentration was found high in the range of 3.54 mg/kg to 20.86 mg/kg. The variation of results might possibly be attributed to the soil type, redox condition and excessive usage of fertilizers and pesticides [24,25,26,27]. Additionally, the Pb existing naturally in the soil, the emissions from vehicles and contaminated irrigation water by industrial waste may have caused the high Pb concentration [28]. However, statistically, there were significant differences at *p* < 0.05 for Cu, Cd, Cr, Pb and As of paddy soil samples from all sampling areas.

### 3.2. Bio-Accessibility of Heavy Metals

The mean for bio-accessibility of heavy metal concentrations in paddy grain samples from Sabak Bernam, Sekinchan, Tanjung Karang and Besut are shown in Table 2. The highest bio-accessibility of heavy metal concentrations found in paddy grains was Cr, followed by Cu, Pb, As and Cd. Based on one sample *T*-test, there were significant differences among all bio-accessibility heavy metal concentrations in all paddy grain samples (*p* values < 0.05). All bio-accessibility concentrations of heavy metals in the paddy grain studied fell below the maximum permissible limit prescribed by the Malaysian Food Regulations 1985 [15] and FAO/WHO CAC 1984 [14] except for heavy metal elements of Pb in Sabak Bernam plot 3 (SBP3) sample, which was in the range of 5.022 ± 0.20 mg/kg and Cr in paddy grain samples for all areas involved.

Kabata-Pendias and Pendias [26] recommended the safe concentration limit for Cr in paddy plants by 30 mg/kg while Kitagishi and Yamane [29] suggested an acceptable limit of concentration for Cr in upper sections of paddy plants cultivated in Japan by 35 mg/kg. The ranking order of bio-accessibility of heavy metals in paddy grains of Sabak Bernam, Sekinchan, Tanjung Karang and Besut was Pb > Cr > Cu > As > Cd suggesting that lead (Pb) and chromium (Cr) were the most abundant heavy metals in all paddy grain samples. The concentration of Cr and Pb in rice grain of all studied areas were found to be high and corroborated with previous studies that showed elevated levels of Cd and Pb in rice [24,25]. Therefore, a control measure should be taken by responsible authorities in order to control the heavy metal pollution by strictly guiding the proper usage of fertilizer and pesticide during cultivation activities.

In Malaysia, governments have three additional responsibilities related to the establishment of food controls. First, they should conduct research into testing and evaluation methods for determining the safety of food ingredients and processes. Governments need to have a good research base because food controls should only be imposed based on a sound scientific basis. Second, governments need to audit industry performance to ensure that companies are complying with standards and that standards are being uniformly applied. This involves training inspection personnel so that they have a good understanding of the technologies and processes involved, as well as conducting inspections in an even-handed and fair manner. Third, governments must communicate with industries and consumers about food controls. It is important that all affected industry members know their obligations so that they can comply [15].

### 3.3. Health Risk Assessment Application

**Figure 1:**
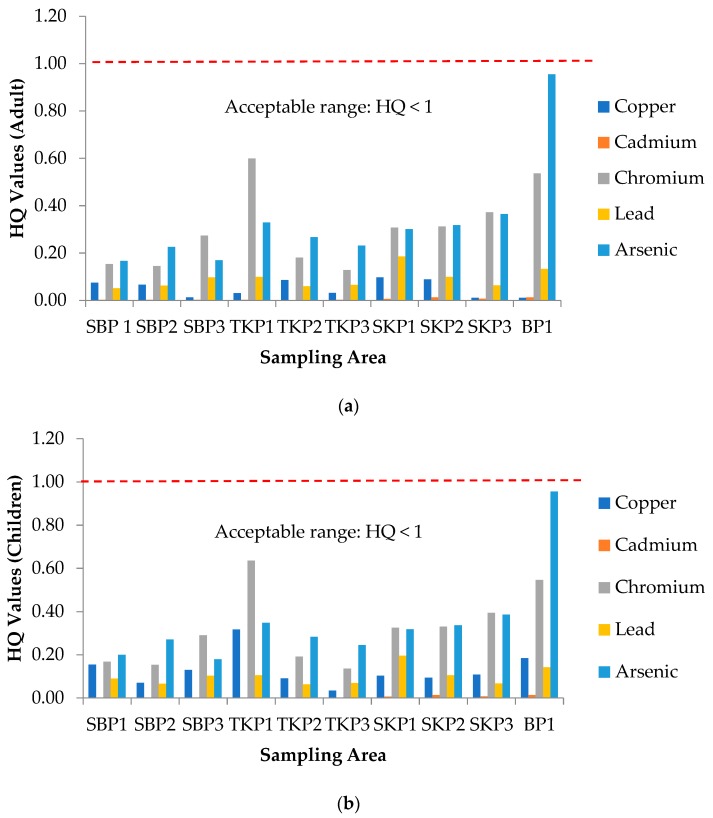
Hazard Quotient (HQ) values for non-carcinogenic health risk for (**a**) adults and (**b**) children in 10 different plot area samples. SBP1: Sabak Bernam Plot 1, SBP2: Sabak Bernam Plot 2, SBP3: Sabak Bernam Plot 3, TKP1: Tanjung Karang Plot 1, TKP2: Tanjung Karang Plot 2, TKP3: Tanjung Karang Plot 3, SKP1: Sekinchan Plot 1, SKP2: Sekinchan Plot 2, SKP3: Sekinchan Plot 3, and BP1: Besut Plot 1.

**Figure 2:**
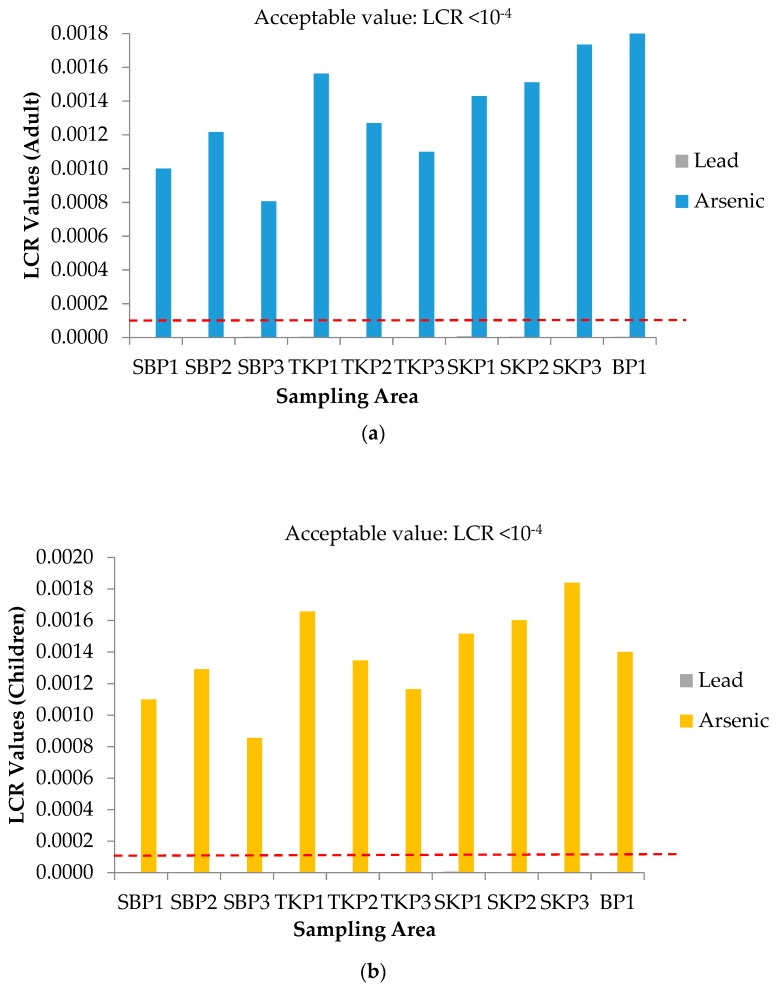
Lifetime Cancer Risk (LCR) values for carcinogenic health risk for (**a**) adults and (**b**) children in 10 different plot area samples. SBP1: Sabak Bernam Plot 1, SBP2: Sabak Bernam Plot 2, SBP3: Sabak Bernam Plot 3, TKP1: Tanjung Karang Plot 1, TKP2: Tanjung Karang Plot 2, TKP3: Tanjung Karang Plot 3, SKP1: Sekinchan Plot 1, SKP2: Sekinchan Plot 2, SKP3: Sekinchan Plot 3, and BP1: Besut Plot 1.

Health risk assessment involves the determination of non-carcinogenic and carcinogenic health risks. For the determination of non-carcinogenic health risk, the hazard quotient (HQ) values for single heavy metals exposure to adults and children are presented in Figure 1a,b, respectively. All HQ values for single heavy metals exposure for adults and children were less than 1 (HQ < 1), which indicated that there was no potential for non-carcinogenic health risk. The results were in line with previous studies by Omar et al. [7]. Meanwhile, for the determination of carcinogenic health risk, Figure 2a,b represents the lifetime cancer risk (LCR) values of adults and children for three plots of Sekinchan, Tanjung Karang, Sabak Bernam and one plot of Besut, respectively. According to USEPA, the cancer slope factor (CSF) values of the Cu element was not available to measure the LCR values in this study. Additionally, Cu was regarded as a non-carcinogenic element [27]. The acceptable range values recommended by USEPA [12] were in the range of 1 × 10^−6^ to 1 × 10^−4^, which meant that there was a one to one hundred in a million chance of additional human cancer over a 70-year lifetime that was considered as an acceptable or inconsequential risk.

Figure 2a,b showed that all LCR values for single heavy metal arsenic (As) exposure of adults and children were higher than the acceptable LCR values recommended by USEPA (LCR > 1 × 10^−4^) [12], indicating a high potential carcinogenic risk from rice consumption. Fu et al. [30] suggested that there is greater LCR value for arsenic (As), which indicates it is present only in the form of inorganic As and 100% of bio-accessibility for a consumer. However, the carcinogenic risk of arsenic (As) may be overestimated since the percentage of inorganic As does not constitute 100% in food materials [31]. In addition, the LCR values of single lead (Pb) exposure in adults (range from 2.0 × 10^−6^ to 6.0 × 10^−6^) and children (range from 0 to 1.0 × 10^−5^) were lower than the acceptable values recommended by USEPA [12]. The LCR values of single As and Pb exposure of adults and children were supported by the results obtained from the studies done by Praveena and Omar [7]. However, the total Lifetime Cancer Risk (LCR_Total_) values for As and Pb (Table 3) concluded that there was a chance of getting cancer through the exposure of As and Pb to adults and children (LCR_Total_ > 1 × 10^−4^).

This study provides the current trend of heavy metal concentrations found in paddy soil and grains samples from areas of Selangor and Terengganu, Malaysia. This trend shows that environmental contaminants, food safety and security and human health are inextricably linked. Overall, the assessment conducted only measured the intake of toxic heavy metals from rice consumption. Other pathways of heavy metal intake such as the consumption of contaminated vegetables, fruits, fish, meat, water and milk including dust inhalation and dermal contact may also influence human exposure [32,33]. There was the limitation that arose from conducting HRA where the non-carcinogenic and carcinogenic health risks estimation for rice grain needs further investigation since the values used were adopted from previous reports and restricted to certain limitations. For example, the ingestion rate (IR) for Malaysians in the present study was obtained from the study conducted by Zheng et al. [33] and there was no data consumption specifically for the Selangor population. Therefore, a complete and valid questionnaire is needed to obtain the actual Malaysian ingestion rate (IR) and body weight (BW) values to be used in such an assessment.

## 4. Conclusions

The findings conclude that lead (Pb) and chromium (Cr) were the most abundant heavy metal concentrations found in the paddy soil samples contributing to the highest bio-accessibility heavy metal concentrations of Cr in paddy grains followed by Cu, Pb, As and Cd. The total Hazard Quotient (HQ_Total_) values for bio-accessibility of heavy metal exposure of adults and children were less than one indicating that there were no non-carcinogenic health risks present. On the other hand, the total Lifetime Cancer Risk (LCR_Total_) values exceeded the acceptable value (LCR_Total_ > 1 × 10^−4^) indicating that both adults and children may experience some adverse health effects in the future after rice ingestion. Health risk assessment indicated high potential of non-carcinogenic risk and carcinogenic risk especially for the As element, which was observed in adult and children exposures. These findings suggested that a control measure on paddy grain from Sabak Bernam, Sekinchan, Tanjung Karang and Besut must be implemented and strengthened to ensure that the risk of heavy metals exposure reduces especially among children as their growth development could be interrupted by the toxic effects of heavy metals.

However, the exposure of heavy metals was measured from the raw rice grain, which does not undergo further processing as commercial rice in the market. Hence, health risk assessment should be further investigated since the study output can be overestimated by the variation, level and distribution of heavy metals in rice grain that has been cultivated. In future, the study related to heavy metal assessment on paddy soils and rice grain should be continued by examining physical–chemical characteristics such as pH of soils, organic matter level and the size of the rice grain. The assessment on fertilizer used for paddy cultivation can be included also in order to identify the primary reason for heavy metal contamination.

## Figures and Tables

**Table 1:** Concentrations of heavy metal in paddy soil samples (mg/kg-dry).

Plot Area/Sampling Area		Mean Concentration ± SD (mg/kg)				
		Copper (Cu)	Cadmium (Cd)	Chromium (Cr)	Lead (Pb)	Arsenic (As)
Sabak Bernam	Plot 1	0.442 ± 0.18	0.015 ± 0.01	1.108 ± 0.03	1.071 ± 0.09	0.345 ± 0.08
	Plot 2	0.498 ± 0.11	0.012 ± 0.00	0.857 ± 0.06	1.100 ± 0.04	0.365 ± 0.07
	Plot 3	0.413 ± 0.02	0.008 ± 0.00	0.745 ± 0.08	0.781 ± 0.03	0.283 ± 0.05
Tanjung Karang	Plot 1	0.611 ± 0.03	0.014 ± 0.01	0.859 ± 0.10	1.084 ± 0.02	0.413 ± 0.01
	Plot 2	0.456 ± 0.05	0.013 ± 0.00	0.870 ± 0.06	1.197 ± 0.09	0.490 ± 0.03
	Plot 3	0.547 ± 0.04	0.014 ± 0.00	0.965 ± 0.08	0.984 ± 0.03	0.380 ± 0.03
Sekinchan	Plot 1	0.601 ± 0.04	0.022 ± 0.00	1.064 ± 0.10	1.508 ± 0.03	0.385 ± 0.05
	Plot 2	0.618 ± 0.06	0.017 ± 0.00	1.157 ± 0.05	1.703 ± 0.10	0.529 ± 0.04
	Plot 3	0.468 ± 0.03	0.010 ± 0.00	0.825 ± 0.09	1.083 ± 0.06	0.357 ± 0.02
Besut	Plot 1	0.801 ± 0.03	0.034 ± 0.01	0.678 ± 0.06	2.510 ± 0.13	0.210 ± 0.02
*p* Values		0.000	0.000	0.000	0.000	0.000
Comparison with:						
Dutch Soil Remediation Circular 2009		190	13.0	NA	530.0	76.0
European Standards Agriculture Soils		140	3	NA	300	NA

NA = not available; * Significant level at *p* < 0.05 for one sample *T*-test.

**Table 2:** Bio-accessibility of heavy metal concentrations in paddy grain samples (mg/kg-dry).

Plot Area/Sampling Area		Mean Concentration ± SD (mg/kg)				
		Copper (Cu)	Cadmium (Cd)	Chromium (Cr)	Lead (Pb)	Arsenic (As)
Sabak Bernam	Plot 1	0.005 ± 0.08	0.013 ± 0.00	3.430 ± 0.10	0.951 ± 0.02	0.541 ± 0.00
	Plot 2	1.703 ± 0.04	0.010 ± 0.00	5.106 ± 0.07	0.762 ± 0.02	0.417 ± 0.01
	Plot 3	1.790 ± 0.05	0.054 ± 0.00	5.238 ± 0.05	5.022 ± 0.20	0.250 ± 0.00
Tanjung Karang	Plot 1	1.538 ± 0.00	0.015 ± 0.00	4.979 ± 0.16	0.501 ± 0.02	0.236 ± 0.01
	Plot 2	1.407 ± 0.02	0.954 ± 0.02	5.190 ± 0.20	0.352 ± 0.01	0.230 ± 0.00
	Plot 3	1.488 ± 0.05	0.002 ± 0.00	5.623 ± 0.12	0.365 ± 0.03	0.244 ± 0.01
Sekinchan	Plot 1	1.258 ± 0.01	0.006 ± 0.00	4.496 ± 0.10	0.251 ± 0.01	0.327 ± 0.01
	Plot 2	1.351 ± 0.03	−0.008 ± 0.00	5.181 ± 0.07	0.196 ± 0.01	0.211 ± 0.01
	Plot 3	1.232 ± 0.03	−0.006 ± 0.00	5.414 ± 0.04	1.852 ± 0.13	0.189 ± 0.00
Besut	Plot 1	1.866 ± 0.02	0.139 ± 0.01	5.463 ± 0.01	0.422 ± 0.01	0.256 ± 0.01
*p* Values		0.006	0.000	0.001	0.000	0.000
Comparison with:						
Malaysian Food Regulations 1985		30	1.0	NA	2.0	1.0
FAO/WHO CAC 1984		10	0.4	1.0	0.2	1.4

NA = not available; * Significant level at *p* < 0.05 for one sample *T*-test.

**Table 3:** Total Hazard Quotient (HQ_Total_) values and total Lifetime Cancer Risk (LCR_Total_) values for Cu, Cd, Cr, Pb, As in adults and children.

Plot Area/Sampling Area	HQ_Total_		LCR_Total_	
	Adults	Children	Adults	Children
SBP1	0.5745	0.6091	**0.0015**	**0.0016**
SBP2	0.4441	0.4708	**0.0013**	**0.0014**
SBP3	0.3789	0.3854	**0.0017**	**0.0017**
TKP1	0.8683	0.9211	**0.0025**	**0.0027**
TKP2	0.6078	0.6444	**0.0016**	**0.0017**
TKP3	0.5145	0.5455	**0.0013**	**0.0014**
SKP1	0.7296	0.7736	**0.0020**	**0.0021**
SKP2	0.7478	0.7928	**0.0022**	**0.0023**
SKP3	0.8496	0.9008	**0.0024**	**0.0026**
BP1	0.3815	0.3658	**0.0012**	**0.0012**

Bold values represent LCR_Total_ values that are more than the acceptable range (LCR > 0.0001).
